# Salivary lubricity (*ex vivo*) enhances upon moderate exercise: A pilot study

**DOI:** 10.1016/j.archoralbio.2020.104743

**Published:** 2020-08

**Authors:** Mark Hopkins, Christine Boesch, Matthew Lansdall, Conor Mullen, Alan Mighell, Sue Pavitt, Anwesha Sarkar

**Affiliations:** a Nutritional Sciences and Epidemiology Group, School of Food Science and Nutrition, Faculty of Environment, University of Leeds, Leeds, LS2 9JT, UK; b School of Dentistry, Faculty of Medicine & Health, University of Leeds, Leeds, LS2 9LU, UK; c Food Colloids and Bioprocessing Group, School of Food Science and Nutrition, Faculty of Environment, University of Leeds, Leeds, LS2 9JT, UK

**Keywords:** Saliva, Tribology, MUC5B, α-amylase, Exercise

## Abstract

**Objective:**

This study sought to examine the effects of moderate intensity exercise on lubrication performance of saliva. We hypothesized that exercise would result in enhanced salivary lubricity by direct sympathetic stimulation of the salivary proteins.

**Study design:**

In total, 11 healthy young pre-menopausal female participants (mean age: 24.4 ± 1.8 years, BMI: 22.1 ± 1.9 kg/m^2^) were included in a within-subjects repeated measures experimental design. Unstimulated whole saliva was collected at rest (S_0_), immediately after 45 min of moderate intensity cycling at ∼70 % maximum heart rate (mean: 133.4 ± 0.8 bpm) or time-match quiet rest (S_1_), and after a 60 min of recovery period (S_2_). *Ex vivo* salivary lubricity were measured using soft tribology. Total protein content, mucin (MUC5B) concentration, and *α*-amylase activity were determined.

**Results:**

Tribology results revealed that moderate intensity exercise resulted in enhanced lubricity of saliva with an order-of-magnitude lower friction coefficients in the boundary regime at S_1_ and S_2,_ with frictional forces being significantly lower at S_1_ (*p < 0.001*) and S_2_ (*p < 0.001*) as compared to the Control procedure. Total protein and *α*-amylase secretion also increased in the Exercise procedure at S_1_ (*p < 0.05*), but concentrations returned to baseline levels at S_2_.

**Conclusions:**

Moderate intensity exercise leads to an increase in *α*-amylase and total protein secretion resulting in enhanced lubrication performance of the saliva. However, the lubrication performance was not related to MUC5B content, suggesting the role of other proteinaceous species acting as lubricants. This proof-of-concept study serves as the first step to design exercise interventions in populations with dry mouth conditions.

## Highlights

• Moderate intensity exercise leads to enhanced lubrication performance of saliva.
• Such enhanced lubrication performance was sustained after 60 min of rest.
• Protein content and *α*-amylase activity in saliva was elevated post-exercise.
• Protein content and *α*-amylase activity returned to baseline with an hour.
• Effects of exercise on salivary mucin (MUC5B) content was not observed.

## 1 Introduction

Saliva is a nature-engineered lubricant found in the oral cavity and is fundamental to eating, swallowing, speech and one’s daily functioning. Recently, there has been an escalation in research interest on salivary lubrication largely fueled by the increased incidence of dry mouth or xerostomia resulting in poor quality and quantity of saliva (Guggenheimer & Moore, 2003; Xu, Laguna, & Sarkar, 2019). Xerostomia increases the risk of dental caries, periodontal disease, candidiasis, oral ulceration, dysphagia, all of which can adversely impact nutritional status and quality of life (Guggenheimer & Moore, 2003). Renewed research interests in finding optimal therapies to treat lubrication failure of saliva is driven by increased incidence of head and neck cancers and associated radiation therapy, rising prevalence of systemic autoimmune Sjögren's syndrome and use of polypharmacy coupled with a growing ageing population. The current topical therapies (*e.g.* sprays, hydrogels) such as carboxymethyl cellulose, xanthan gum (Nieuw Amerongen & Veerman, 2003; Oh, Lee, Kim, & Kho, 2008), that are far from the composition of real human saliva bring only limited symptomatic relief and any benefits are often short-lived (Vinke, Kaper, Vissink, & Sharma, 2018). This is in part because, in designing most substitutes, rheology (resistance to flow or viscosity) has been considered as the “gold standard” characteristic with the goal to match the viscosity of human saliva (Sarkar, Xu, & Lee, 2019; Xu et al., 2020), but fails to consider the tribological (lubrication) aspects of saliva, which is the main focus of this paper.

Although saliva is largely an aqueous fluid containing 99 % water, it is the proteins (*i.e.* mucins (MUC5B) and other low molecular weight proteins) and ions contributing to the rest of the salivary composition that renders saliva its unique rheological (viscosity, elasticity, stickiness) and lubrication properties (Humphrey & Williamson, 2001; Sarkar, Kanti, Gulotta, Murray, & Zhang, 2017; Sarkar, Ye, & Singh, 2017; Sarkar, Xu et al., 2019). Physiologically, the secretion of saliva can be controlled through two separate pathways: the sympathetic and parasympathetic nervous systems, producing varying compositional characteristics in saliva (Garrett, 1987; Proctor & Carpenter, 2014). Generally it is hypothesized that when the sympathetic nervous system is stimulated, salivary protein secretion is increased, while innervation of the parasympathetic nervous system results in evoking greater salivary volume or water content in saliva. Despite the progress made towards our understanding of salivary regulation (Proctor & Carpenter, 2007), it is not yet completely understood. Physical exercise is well-recognized to innervate the sympathetic nervous system (Paterson, 1996), affecting salivary composition and consequently its material properties. For instance, exercise has been found to stimulate the hypothalamic pituitary-adrenal and sympathetic-adreno-medullary pathways, resulting in higher concentrations of *α*-amylase being secreted in saliva (Koibuchi & Suzuki, 2014). Recently, moderate exercise has been demonstrated to be effective in not only increasing salivary protein, MUC5B and salivary flow rates (Ligtenberg, Brand, van den Keijbus, & Veerman, 2015), but also in improving properties such as salivary viscosity (Ligtenberg, Liem, Brand, & Veerman, 2016). However, whether such exercise-induced increase in protein concentration can also improve lubricity of saliva remains elusive in literature to date. Salivary lubrication has been studied in the literature using soft tribological approaches *i.e.* measuring friction coefficients using polymer-polymer contact surfaces representing proxies for tongue-palate surfaces (Bongaerts, Rossetti, & Stokes, 2007; Xu et al., 2020). Therefore, measuring friction coefficients after exercise can serve as a novel dimension to understand how exercise affects lubricity of saliva and provide the first piece of evidence of exercise being a potential strategy to address dry mouth problems.

The aim of this pilot study was to examine changes in salivary lubricity after a bout of moderate intensity cycling for 45 min in healthy females as compared to a time-matched rest period. It was hypothesized that exercise would induce enhancement in salivary lubrication performance due to its effects on increases in protein and MUC5B content.

## 2 Materials and methods

### 2.1 Participants

Eleven healthy pre-menopausal female participants with a mean (± SD) age of 24.4 ± 1.8 years and mean BMI of 22.1 ± 1.9 kg/m^2^ were enrolled in this study (see Table 1). Participants were recruited using emails, posters and social media advertisements. Potential participants were screened and included if they were non-smokers, had no self-reported systemic or oral diseases, were over the age of 18 years, not under any medication affecting metabolism or salivation (with the exception of oral contraception), and did not display any counter-indications for exercise (Physical Activity Readiness Questionnaire) (Thomas, Reading, & Shephard, 1992). This study was approved by the Faculty Research Ethics Committee at the University of Leeds (ethics reference: MEEC 16-046).

**Table 1:** Descriptive characteristics of the participants.

Characteristics	Mean ± SD
Age (years)	24.4 ± 1.8
BMI (kg/m^2^)	22.1 ± 1.9
Height (cm)	164.9 ± 5.5
Weight (kg)	60.9 ± 8.3
70 % Max heart rate (bpm)	133.3 ± 0.8

### 2.2 Experimental design

**Fig. 1:**
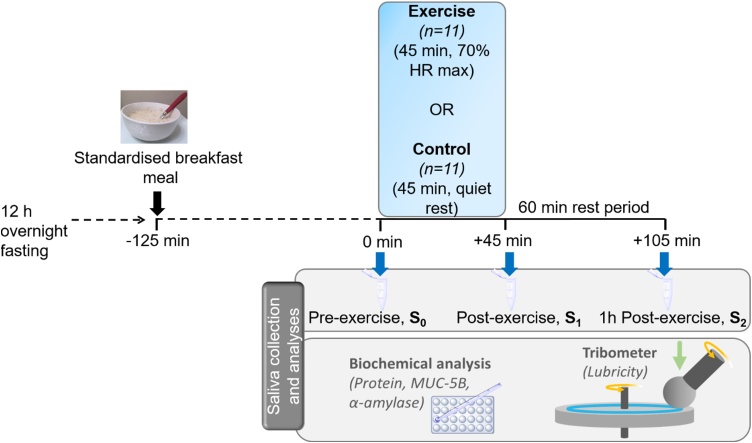
Schematic overview of the experimental design, where n represents the number of participants.

A within-subjects repeated measures experimental design was used in which participants completed ‘Experimental’ (45 min of exercise – specifically cycling at 70 % maximum heart rate (HR_max_)) and ‘Control’ (time-matched rest comprising quiet sitting) procedures in randomized order (see Fig. 1). There was a minimum of three days wash-out period between the delivery of the second procedure. To exclude any residual effects of previous exercise, participants were asked to refrain from any strenuous exercise for 24 h before each procedure. Experimental and Control procedures were performed at the same time of the day to exclude diurnal variations.

As shown in Fig. 1, participants were given a standardized porridge pot (Tesco Original, UK; 207 kcals, 3.31 g fat, 34.42 g carbohydrates, 7.91 g protein) to consume in their home environment at 08:00 am on the day of the Experimental and Control procedures. Participants were asked to arrive at the laboratory half an hour before each procedure started (approximately 10:00 a.m.), with body weight measured to the nearest 0.1 kg after voiding (Seca 763, Seca Birmingham, UK) and height measured to the nearest 0.5 cm using a portable stadiometer (Seca Portable height measure, Leicester, UK). Participants were then fitted with a heart rate monitor (Polar A300, Polar, Warwick, UK) around the sternum and a wrist worn heart rate watch (Polar A300, Polar, Warwick, UK).

During the Experimental procedure, participants performed 45 min of exercise on a cycle ergometer (Watt Bike Trainer, Wattbike, Nottingham) at 70 % HR_max_ (HR_max_ = 208 – (0.7 × age)) (Roy & McCrory, 2015) (mean values provided in Table 1). Exercise was supervised to ensure compliance to the prescribed intensity, and resistance was adjusted to ensure the required workload was maintained throughout. During the Control procedure, participants performed a 45 min period of seated rest period (Hopkins, Blundell, & King, 2014). Following the 45 min period of exercise or rest, participants were asked to remain in the laboratory and complete a further 60 min recovery period consisting of quiet rest. Throughout the exercise and rest protocols, participants were asked to refrain from ingesting any fluids. During both procedures, unstimulated saliva samples were collected immediately before the 45 min period of exercise or rest (S_0_), immediately after (S_1_) and following the 60 min recovery period (S_2;_ Fig. 1). Noteworthy, that both the Exercise and Control conditions were carried out at thermo-neutral environment (∼ 22 °C), and therefore losses in body fluid were likely to be modest. However, body weight measurements were not carried out post-exercise so the degree of body fluid loss thus is unknown. Hence, the effect of fluid loss during exercise on salivary properties remain as a limitation in this study.

### 2.3 Saliva collection

Unstimulated whole saliva (2 mL) was collected according to a previous study (Navazesh, 1993) by expectoration into a chilled amber-colored polypropylene tube during 5 min. Hereafter, an equal volume of 2 mM phosphate buffer, pH 6.8, was added, and the saliva was homogenized for 20 s using a vortex mixer. Saliva was centrifuged for 3 min at 4000 × g to remove debris; it is worth noting that centrifugation does not influence the lubrication properties of saliva (Zhang, Zheng, Zheng, & Zhou, 2016). The supernatant was further diluted for tribological analysis (see below) carried out on the same day and three separate aliquots (250 μL each) were stored at −20 °C until further use for total protein, α-amylase and MUC5B assays, respectively.

### 2.4 Oral tribology measurements

Oral tribology measurements of saliva (*ex vivo*) were conducted with slight adaptation of the well-established method described previously (Krop, Hetherington, Holmes, Miquel, & Sarkar, 2019; Laguna, Farrell, Bryant, Morina, & Sarkar, 2017) using ball-on-disc set up in a Mini Traction Machine (MTM2 tribometer, PCS Instruments, London, UK). Freshly collected saliva at different time points (S_0_, S_1_ and S_2_) in both Control and Experimental procedures for each of the eleven participants were diluted in buffer (1:1 v/v), centrifuged and the supernatant was further diluted with buffer to make it to 9 mL (*i.e.* 16 vol% unstimulated whole human saliva) for tribological analyses on the same day. The final sample dilution of saliva was Commercially available polydimethylsiloxane (PDMS) ball (diameter of 4 mm, MTM ball Slygard 184, 50 Duro, PCS Instruments, London, UK) and disc (diameter of 46 mm, thickness of 4 mm, MTM ball Slygard 184, 50 Duro, PCS Instruments, London, UK) were used as surfaces to mimic tongue and palate for the oral tribology measurements (surface roughness of the PDMS tribopairs, R_a_ < 50 nm). The saliva supernatant (9 mL) was loaded into the minipot equipped with the PDMS ball and disc, where these tribopairs were rotated at different speeds to create a relative motion between the surface of the ball and the disc, resulting in a slide-to-roll ratio (SRR) of 50 %, and the temperature was maintained at 37 °C, simulating oral procedures. The entrainment speed was calculated as the average velocity of the two contacting surfaces (*i.e.* ball and disc). The entrainment speed was reduced from 300 to 1 mm/s to simulate tongue movement, and friction forces were measured at a load of 2 N with a maximum of 200 kPa of Hertzian contact pressure (Sarkar, Andablo-Reyes, Bryant, Dowson, & Neville, 2019). Six curves of friction coefficients *versus* entrainment speeds were plotted for each participant by averaging values for both Control and Experimental procedures for S_0_, S_1_ and S_2_ time points. Friction forces at entrainment speeds of 3 mm/s and 50 mm/s were compared, which represented the boundary and mixed lubrication regimes, respectively (Krop et al., 2019).

### 2.5 Biochemical assays

Separate aliquots of saliva were used for each assay, which were carefully defrosted on ice and then briefly centrifuged. Supernatants (*i.e.* 50 vol% unstimulated whole human saliva) were assayed for total protein using BCA assay (Pierce, Fisher Scientific, Loughborough, UK) and the results were compared to a standard curve generated with bovine serum albumin. Salivary mucin (MUC5B) was determined by a commercially available ELISA assay (MUC5B ELISA Kit, Aviva Systems Biology, Insight Biotechnology, Wembley, UK). The Salimetrics *α*-amylase kit (Stratech, Ely, UK) was used to measure salivary *α*-amylase enzyme activity. All analyses were run in duplicate and absorbance values recorded using Tecan Spark 10 M microplate reader (Tecan, Reading, UK). Results were expressed as Units (amylase) or ng (MUC5B) per mL saliva and mg protein.

### 2.6 Statistical analyses

Tribology and results from biochemical assays are displayed as means and standard deviations for Experimental and Control procedures for the three time points (S_0_, S_1_ and S_2_). Repeated measures two-way analysis of variance (time* procedure) tests were conducted to examine differences between Control and Exercise procedures for tribology data, total protein, α-amylase and MUC5B. Where appropriate, Greenhouse-Geisser probability levels were used to adjust for sphericity, and paired *t*-test *post-hoc* comparisons were used if statistical significance was detected. Alongside *p*-values, effect sizes are reported as partial eta-squared (ƞ_p_^2^) for ANOVA models. Pearson correlations were performed between S_0_ and S_1_ boundary and mixed regime saliva lubricity and total protein, *α*-amylase and MUC5B in the Experimental procedure (*e.g.* pre to post exercise changes). A correlation *r* value of 0.2 was interpreted as a small effect, 0.5 as a medium effect and 0.8 as a large effect (Sullivan & Feinn, 2012). All statistical analyses were conducted using SPSS (IBM SPSS, Chicago, Illinois, Version 26) and a significance value was considered at *p < 0.05*.

## 3 Results

### 3.1 Salivary lubricity

**Fig. 2:**
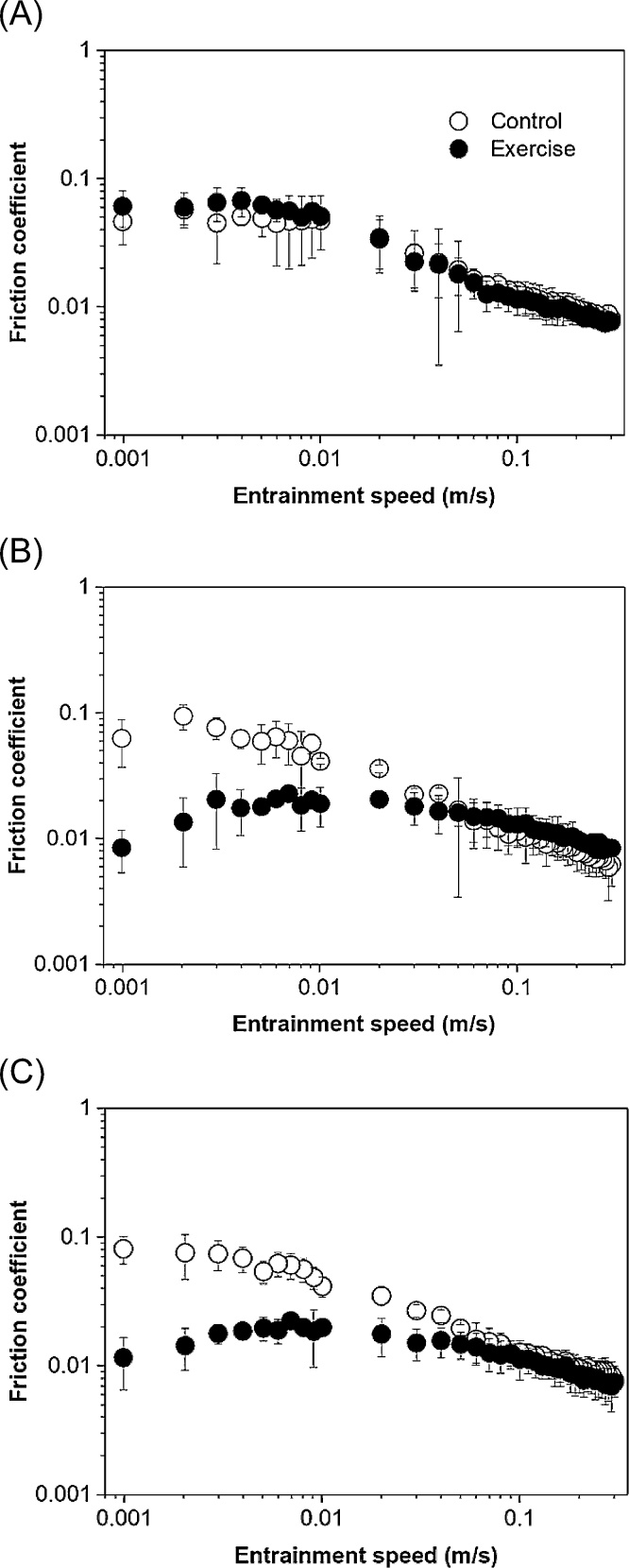
Mean friction coefficients of saliva of participants as a function of entrainment speed before exercise (S_0_, panel A), immediately post-exercise (S_1_ panel B) and 1 h post-exercise (S_2_, panel C) in the Control procedure (open symbols) and Experimental procedure (closed symbols).Error bars represent standard deviations (n = 11).

To compare the friction coefficients of saliva (*ex vivo*) collected from the Experimental and the Control groups, respectively, at different time points (S_0_, S_1_ and S_2_) (Fig. 2**A–C**), a ball-on-disc tribometer with PDMS tribopairs was used. Although the hydrophobicity, surface roughness and modulus of PDMS surfaces do not exactly mimic the human tongue-palate surfaces (Sarkar, Kanti et al., 2017; Sarkar, Ye et al., 2017; Sarkar, Andablo-Reyes et al., 2019), PDMS serves as the closest approximation to oral surfaces among the available polymeric and metallic surfaces to date. Interestingly, irrespective of the Experimental or the Control procedures, Fig. 2 showed similar pattern in the evolution of friction coefficients as a function of entrainment speed. In general, all friction curves showed a boundary regime where the friction coefficients were independent of the entrainment speeds (0.003−0.01 m/s) followed by a clear onset of mixed regime where the friction coefficients decreased significantly < 0.1 with increasing speeds.

**Fig. 3:**
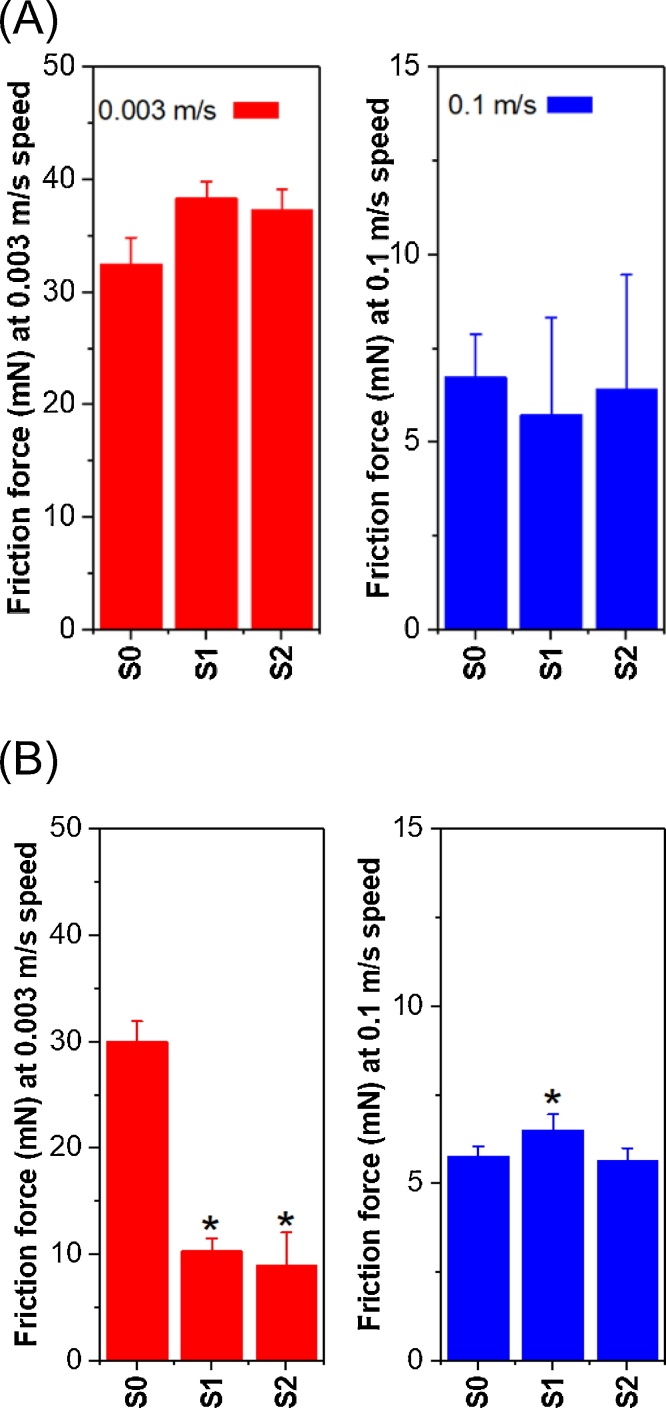
Mean friction force (mN) of saliva at boundary regime (0.003 m/s entrainment speed) and mixed regime (0.1 m/s entrainment speed) of Control procedure (A) and Experimental procedure (B) before exercise (S_0_), immediately post-exercise (S_1_) and 1 h post-exercise (S_2_). Error bars represent standard deviations (n = 11). Statistical diﬀ ;erences were subject to analysis of variance (ANOVA) with repeated measures and paired *t*-test *post-hoc* comparisons. *significant difference between the Experimental and Control conditions at S_1_ and S_2_ (*p < 0.05*).

As one might expect, Fig. 2**A** demonstrates a perfect overlap between the saliva from the participants in the Control and Exercise procedures since at time point S_0_, saliva was collected before the exercise intervention commenced. This confirms feasibility of the soft tribology approach to measure salivary lubricity in line with previous reports (Bongaerts et al., 2007; Sarkar, Xu et al., 2019; Torres et al., 2019; Xu et al., 2020). Frictional forces in the Control and Experimental procedure for the saliva boundary (0.003 m/s) and mixed regimes (0.1 m/s) can be seen in Fig. 3**A** and **B**, respectively. In the boundary regime (0.003 m/s), a significant main effect of time (F_(2, 20)_ = 24.417; *p* < 0.001; ƞ_p_^2^ = 0.709), procedure (F_(1, 10)_ = 289.887; *p* < 0.001; ƞ_p_^2^ = 0.967) and time*procedure interaction (F_(2, 20)_ = 106.276; *p* < 0.001; ƞ_p_^2^ = 0.917) was observed. There were no significant differences in frictional force between the Control and Experimental procedures at S_0_ (*e.g.* baseline), but the frictional force was significantly lower at S_1_ (−28.3 ± 4.9 mN; *p* < 0.001) and S_2_ (−28.5 ± 6.7 mN; *p* < 0.001) in the Experimental procedure as compared to the Control procedure (Fig. 3B). For the post procedure time point (S_1_) (Fig. 2B), the saliva from the Experimental procedure was observed to reduce the friction coefficient by an order of magnitude as compared to the Control samples in the boundary regime. Even after 60 min of rest, the reduction in frictional force in the saliva boundary regime in the Experimental procedure persisted (Fig. 2C), with the boundary friction force at S_2_ remaining significantly lower than S_0_ in the Experimental procedure (−21.2 ± 4.2 mN, *p* < 0.001; Fig. 3B).

Although the boundary friction force was much lower after the Experimental procedure (Fig. 3B), no main effect of time (F_(2, 20)_ = 0.468; *p* = 0.633; ƞ_p_^2^ = 0.045) or procedure (F_(2, 20)_ = 1.177; *p* = 0.303; ƞ_p_^2^ = 0.105) was observed in the mixed regime. There was a significant time*procedure interaction (F_(2, 20)_ = 4.425; *p* = 0.026; ƞ_p_^2^ = 0.307), such that the friction coefficient was 0.78 ± 0.79 mN higher at S_1_ in the Experimental *versus* Control procedure (*p* = 0.008). Despite this small difference in frictional force at S_1_, these data indicate there was no meaningful difference in the frictional behavior in the mixed regime between the Control and Experimental procedures (Fig. 3). These findings suggest exercise increased the boundary lubrication properties of saliva when compared to that of the Control procedure and such lubricity sustained even after an hour of recovery period post exercise, which has never been reported in literature to date.

### 3.2 Total protein, α-amylase and MUC5B

**Fig. 4:**
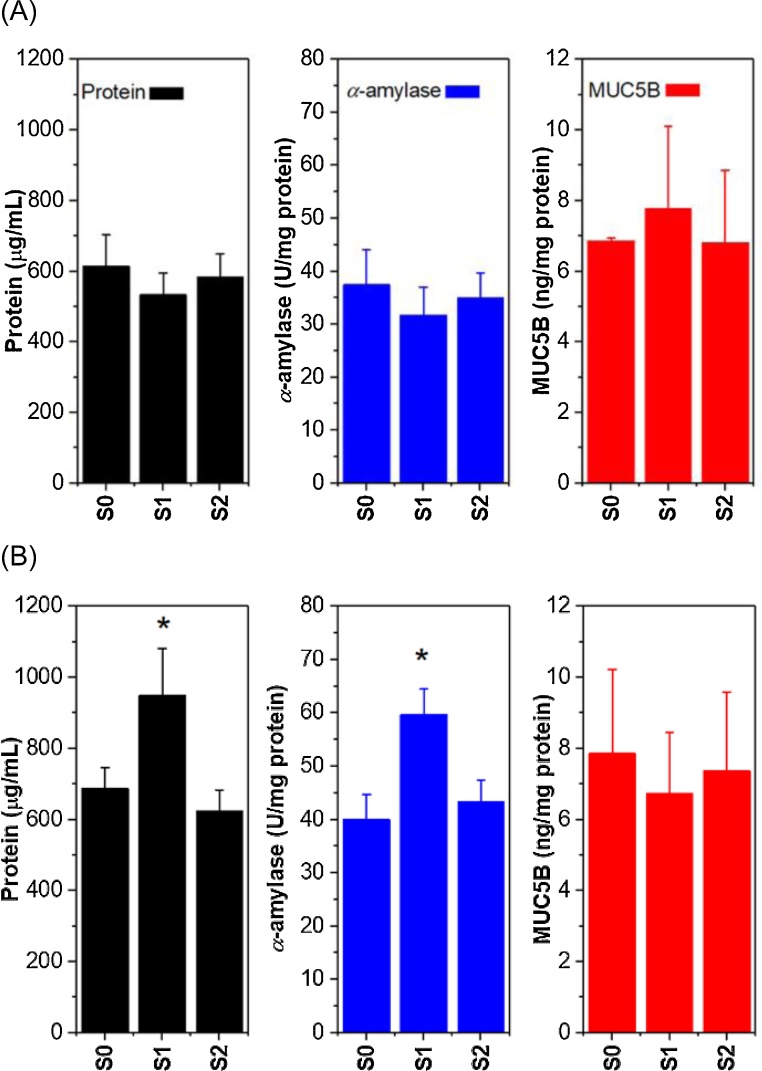
Mean protein (μg/mL), amylase (U/mg protein) and MUC5B (ng/mg protein) in saliva of Control procedure (A) and Experimental procedure (B) before exercise (S_0_), immediately post-exercise (S_1_) and 1 h post-exercise (S_2_). Error bars represent standard deviations, respectively (n = 11). Statistical diﬀ ;erences were subject to two-way analysis of variance (ANOVA) with repeated measures and paired *t*-test *post-hoc* comparisons. *significant difference between S_0_ and S_1_ in the Experimental condition (*p < 0.05*).

In order to gain biochemical understanding behind such boundary salivary lubrication, total protein, *α*-amylase and MUC5B content in the saliva from the two procedures at three different time points were assessed (Fig. 4). In line with the tribology results (Figs. 2**A**–**C** and 3 **A**) salivary protein concentration, *α*-amylase activity and MUC5B remained the same in the Control procedure irrespective of time (Fig. 4A). Interestingly, both salivary protein concentration and *α*-amylase activity increased immediately after exercise in the Experimental procedure, but decreased to baseline values after an hour of the recovery period (Fig. 4B). For total protein, there was no significant main effect of time (F_(2, 20)_ = 2.629; *p* = 0.097; ƞ_p_^2^ = 0.208) or procedure (F_(1, 10)_ = 4.204; *p* = 0.067; ƞ_p_^2^ = 0.296), but a significant time* procedure interaction was seen (F_(2, 20)_ = 7.509; *p* = 0.004; ƞ_p_^2^ = 0.429). Total protein was higher post exercise (S_1_) as compared to baseline (S_0_) during the Experimental procedure n (*p* = 0.028). For *α*-amylase, there was a significant main effect of time (F_(2, 20)_ = 3.565; *p* = 0.047; ƞ_p_^2^ = 0.263) and procedure (F_(1, 10)_ = 7.028; *p* = 0.024; ƞ_p_^2^ = 0.413), and a significant time* procedure interaction (F_(2, 20)_ = 11.420; *p* < 0.001; ƞ_p_^2^ = 0.533). *α*-amylase was significantly higher post exercise (S_1_) as compared to baseline (S_0_) during the Experimental procedure (*p* < 0.001). However, salivary MUC5B concentration did not change significantly in response to the moderate intensity exercise used in this study (*p > 0.05*), with no main effect of time (F_(1.3, 13.3)_ = 0.324; *p* = 0.727; ƞ_p_^2^ = 0.031), procedure (F_(1, 10)_ = 0.060; *p* = 0.812; ƞ_p_^2^ = 0.140) or time* procedure interaction seen (F_(2, 20)_ = 1.627; *p* = 0.222; ƞ_p_^2^ = 0.140).

### 3.3 Associations between the exercise-induced change in salivary lubricity and total protein, α-amylase and MUC5B

To examine whether the aforementioned changes in salivary lubricity with exercise were related to protein type and content, Pearson correlations were performed between the exercise-induced changes in saliva friction coefficients (*e.g.* Exercise procedure S_0_ and S_1_) and the corresponding changes in total protein, *α*-amylase and MUC5B concentration. In the case of the boundary regime (0.003 m s^−1^), no statistical associations were seen between the change in frictional force and the change in total protein (*r* = −0.212, *p* = 0.521, R^2^ = 0.045) and *α*-amylase (*r* = 0.044; *p* = 0.045, R^2^ = 0.044). A small to moderate, but non-significant, association was seen with MUC5B (*r* = 0.385, *p* = 0.243, R^2^ = 0.148). For the mixed regime (0.1 m/s), no association was seen between the change in frictional force and the change in MUC5B (*r* = 0.227, *p* = 0.501, R^2^ = 0.052) or change in total protein (*r* = −0.286, *p* = 0.395, R^2^ = 0.082). A small to moderate, non-significant, association was seen between the change in the friction coefficient and *α*-amylase (*r* = −0.338, *p* = 0.309, R^2^ = 0.115).

## 4 Discussion

Composition of saliva after acute bouts of exercise varying in type, duration and intensity have been studied over the last decades (Chicharro, Lucía, Pérez, Vaquero, & Ureña, 1998). Nevertheless, studying the material properties such as viscosity of saliva is a relatively new undertaking in literature. It has been suggested that salivary viscosity increases immediately following high intensity exercise, but these changes are transient, with viscosity returning to baseline values following 30 min of exercise cessation (Ligtenberg et al., 2016). To our knowledge, the current study is the first to investigate the lubrication performance of saliva post exercise and demonstrate a significant enhancement in the salivary lubricity following moderate intensity exercise.

Results demonstrate that moderate intensity exercise increased salivary lubricity measured *ex vivo* when compared to a time matched control procedure. In particular, it appears from this pilot study that exercise improves the boundary, but not mixed regime, lubrication properties of whole human saliva and that the increased lubricity of saliva was sustained 60 min post-exercise. It is worth noting that viscosity describes the bulk property of saliva whereas the lubrication performance particularly in the boundary region is largely described by the surface adsorption properties *i.e.* the ability of saliva to coat the surface of tongue, palate and other mucosal surfaces (Sarkar, Andablo-Reyes et al., 2019). Hence, it is highly likely that the thin film of salivary species of few nanometers (Stokes, Boehm, & Baier, 2013; Xu et al., 2019) that may be retained on the oral surface after an hour as observed in this study might provide insufficient bulk viscosity effect as observed by Ligtenberg et al. (2016). But such salivary film might still be capable of preventing contact between the rubbing oral surfaces (tongue-palate) and reduce the boundary friction as reflected in the present work.

Such increase in salivary lubricity can be associated with the increase in overall protein content on exercise as observed in this study and in line with previous results by Ligtenberg et al. (2015). Although the protein content of saliva in this study was at the lower end of the range typically found in whole human saliva possibly due to measurements in the morning in the present study (Crosley et al., 2009; Sarkar, Xu et al., 2019), the relative increase of total salivary protein upon exercise was 38 %, which lies in between the range of increase observed for saliva collected from participants subjected to moderate to high intensity exercise procedures by Ligtenberg et al. (2015). Such increase of exercise-induced salivary protein content might be attributed to the direct sympathetic stimulation of the salivary glands by plasma catecholamines that can increase significantly above the anaerobic threshold (which denotes the point at which significant blood lactate accumulation is seen with increasing exercise intensity) (Brooks, 1985; Proctor & Carpenter, 2014). It should also be noted that saliva total protein concentrations have been shown to increase following exercise-induced losses in body fluid and dehydration (Walsh, Montague, Callow, & Rowlands, 2004), but body fluid losses during exercise were not measured in the present study. The effect of exercise on salivary *α*-amylase activity appears to be more pronounced at higher exercise intensities (*e.g.* >70 % maximal aerobic capacity), presumably due to greater sympathetic drive as increased physiological stress is known to be well correlated with increased salivary *α*-amylase secretion (Akizuki, Yazaki, Echizenya, & Ohashi, 2014; Koibuchi & Suzuki, 2014; Oliveira et al., 2010). In our study, a good agreement to the previous report (Ligtenberg et al., 2015) on increase of *α*-amylase activity was demonstrated immediately following exercise (∼49 % increase *versus* baseline), confirming the sympathetic innervation induced by the exercise. Furthermore, it has recently been reported that 20 min of hard RPE-based cycling (15 on the Borg rating of perceived exertion scale) resulted in significant increases in *α*-amylase concentrations immediately post-exercise, but concentrations returned to baseline levels 10 min post-exercise (Weiss, Venezia, & Smith, 2019).

The present data suggest that the enhancement in salivary lubricity immediately following exercise might be explained by the increased total protein content, which is largely dependent upon the sympathetic innervation (Proctor & Carpenter, 2014). Interestingly, the MUC5B did not change following exercise in this study, which is in contrast to previous reports (Ligtenberg et al., 2015). Therefore, we hypothesize that non-mucinous proteins might have been predominantly contributing to the lubricity of saliva following exercise as opposed to the MUC5B; the latter is often mooted as the protagonist in salivary lubricity (Yakubov, Macakova, Wilson, Windust, & Stokes, 2015). Other low molecular weight proteins such as lactoferrin, statherins and proline-rich proteins (PRP-1) are known to have superior boundary lubrication properties (Hahn Berg, Lindh, & Arnebrant, 2004; Xu et al., 2020), which might have increased during exercise and require detailed characterization in future studies. In the present data, no significant associations were seen between changes in salivary lubricity and salivary proteins following exercise. However, this pilot study was not sufficiently powered to do so. Small to moderate (albeit, non-significant) associations were seen between changes in salivary lubricity and MUC5B (boundary lubrication) and *α*-amylase (mixed regime), suggesting these relationships should be further explored using adequately powered samples.

Although both proteins and *α*-amylase activity increased immediately after exercise, they both reduced back to the baseline values following the 60 min recovery period in the present study, which suggests that the sustained effect in boundary lubrication properties observed in the tribology results might not be explained solely by the total proteinaceous species. Such sustained lubrication observed in the tribology results might be the effect of hydration lubrication by the ions in the saliva (Jahn & Klein, 2015); electrolytes such as Na^+^, Mg^2+^ are claimed to increase in saliva upon exercise (Chicharro et al., 1999), which needs more detailed investigation in the future.

## 5 Conclusions

The aim of this study was to assess the effect of a single bout of moderate intensity exercise on *ex vivo* lubricity of saliva collected from young female healthy participants and to determine whether the protein type and content can explain the mechanism behind such improvement of lubricity (if any). This proof of concept study used tribology to test the lubricity of saliva as a function of exercise intervention and recovery period, for the first time. Results demonstrate that moderate intensity exercise has significant effects on salivary lubricity measured *ex vivo* when compared to a time matched control procedure. In particular, it appears that exercise improves the boundary lubrication properties of whole human saliva, and that the increased lubricity of saliva was sustained 60 min post-exercise. Future work should investigate various intensities of exercise in a larger sample size and determine i) the relationship between post-exercise changes on salivary lubricity, lubricity kinetics (*i.e.* length of time such change in lubricity lasts) and underlying changes in proteinaceous species (*e.g.* MUC5B*, α*-amylase, lactoferrin, statherins, PRP-1), and ii) whether such exercise-salivary lubricity relationships are valid in vulnerable populations, such as older adults suffering from dry mouth conditions.

## CRediT authorship contribution statement

**Mark Hopkins:** Methodology, Validation, Conceptualization, Data curation, Writing - original draft, Writing - review & editing, Visualization, Supervision. **Christine Boesch:** Methodology, Formal analysis, Writing - review & editing, Validation. **Matthew Lansdall:** Formal analysis, Investigation. **Conor Mullen:** Formal analysis, Investigation. **Alan Mighell:** Conceptulaization, Methodology, Writing - review & editing. **Sue Pavitt:** Conceptulaization, Methodology, Writing - review & editing. **Anwesha Sarkar:** Methodology, Validation, Conceptualization, Data curation, Writing - original draft, Writing - review & editing, Visualization, Supervision, Project administration, Funding acquisition.

## Declarations of Competing Interest

None.
